# A High-Value, Low-Cost Bubble Continuous Positive Airway Pressure System for Low-Resource Settings: Technical Assessment and Initial Case Reports

**DOI:** 10.1371/journal.pone.0053622

**Published:** 2013-01-23

**Authors:** Jocelyn Brown, Heather Machen, Kondwani Kawaza, Zondiwe Mwanza, Suzanne Iniguez, Hans Lang, Alfred Gest, Neil Kennedy, Robert Miros, Rebecca Richards-Kortum, Elizabeth Molyneux, Maria Oden

**Affiliations:** 1 Department of Bioengineering, Rice University, Houston, Texas, United States of America; 2 Department of Pediatric Emergency Medicine, Baylor College of Medicine, Houston, Texas, United States of America; 3 Department of Pediatrics, College of Medicine and Queen Elizabeth Central Hospital, Blantyre, Malawi; 4 Department of Respiratory Care, Texas Children's Hospital, Houston, Texas, United States of America; 5 Department of Pediatrics, College of Medicine and Kamuzu Central Hospital, Lilongwe, Malawi; 6 Department of Pediatrics, Baylor College of Medicine, Houston, Texas, United States of America; 7 3rd Stone Design, San Rafael, California, United States of America; University of Liverpool, United Kingdom

## Abstract

Acute respiratory infections are the leading cause of global child mortality. In the developing world, nasal oxygen therapy is often the only treatment option for babies who are suffering from respiratory distress. Without the added pressure of bubble Continuous Positive Airway Pressure (bCPAP) which helps maintain alveoli open, babies struggle to breathe and can suffer serious complications, and frequently death. A stand-alone bCPAP device can cost $6,000, too expensive for most developing world hospitals. Here, we describe the design and technical evaluation of a new, rugged bCPAP system that can be made in small volume for a cost-of-goods of approximately $350. Moreover, because of its simple design—consumer-grade pumps, medical tubing, and regulators—it requires only the simple replacement of a <$1 diaphragm approximately every 2 years for maintenance. The low-cost bCPAP device delivers pressure and flow equivalent to those of a reference bCPAP system used in the developed world. We describe the initial clinical cases of a child with bronchiolitis and a neonate with respiratory distress who were treated successfully with the new bCPAP device.

## Introduction

Acute respiratory infections are the leading cause of global child mortality [1]. There is an important need for new, cost-effective technologies to treat infants and small children with respiratory distress. This need is most acute in the hours after birth. 20–38% of deaths in the first 48 hours of life are attributed to respiratory failure [2]. Moreover, complications associated with premature birth, often related to breathing problems, are responsible for an additional 30% of neonatal mortality [3]. In the developed world, babies with respiratory distress syndrome (RDS) receive mechanical ventilatory support; but these lifesaving technologies are too expensive and resource-intensive for most of the developing world. As a result, RDS remains one of the most common causes of the 3 million annual neonatal deaths in the developing world [4]. The need is particularly acute on the African continent, which has the second highest number of preterm births [5].

In the developed world, bubble Continuous Positive Airway Pressure (CPAP) is a gentle and effective tool to manage babies in respiratory distress [6]. Hospitals use tubing, wall air, and oxygen to set up bCPAP at the bedside. Pressure is safely and simply regulated by submerging the end of the tubing in a bottle of water. The depth of the tube in the water determines the pressure in the system. Delivering pressurized flow helps prevent alveolar collapse, thus lowering the work of breathing. Unfortunately, even tertiary hospitals in the developing world often do not have access to wall air and oxygen and cannot implement this lifesaving technique. In such settings, an oxygen canister or oxygen concentrator is sometimes used to deliver almost pure oxygen to the baby, but without the added pressure to open the alveoli, babies still struggle to breathe. A stand-alone bCPAP device recently received FDA approval [7]; however, at $6,000, the system is too expensive for many developing world hospitals.

A successful bubble CPAP system for the developing world must have the following traits: (1) Adjustable flow rates, (2) Ability to mix oxygen into the flow stream; (3) Mechanism to control the pressure delivered to the patient; (4) Low cost; (5) Safe; (6) Durable; (7) Easy to use and repair. We have developed a low-cost bubble CPAP system that meets these necessary performance criteria. Here we describe the technical performance of this device and compare it to that of a reference standard bubble CPAP device used in the developed world. We present two case reports illustrating the use of the low-cost bCPAP device to successfully treat a child with bronchiolitis and a neonate with respiratory distress due to congenital pneumonia in a low-resource setting.

## Methods

**Figure 1:**
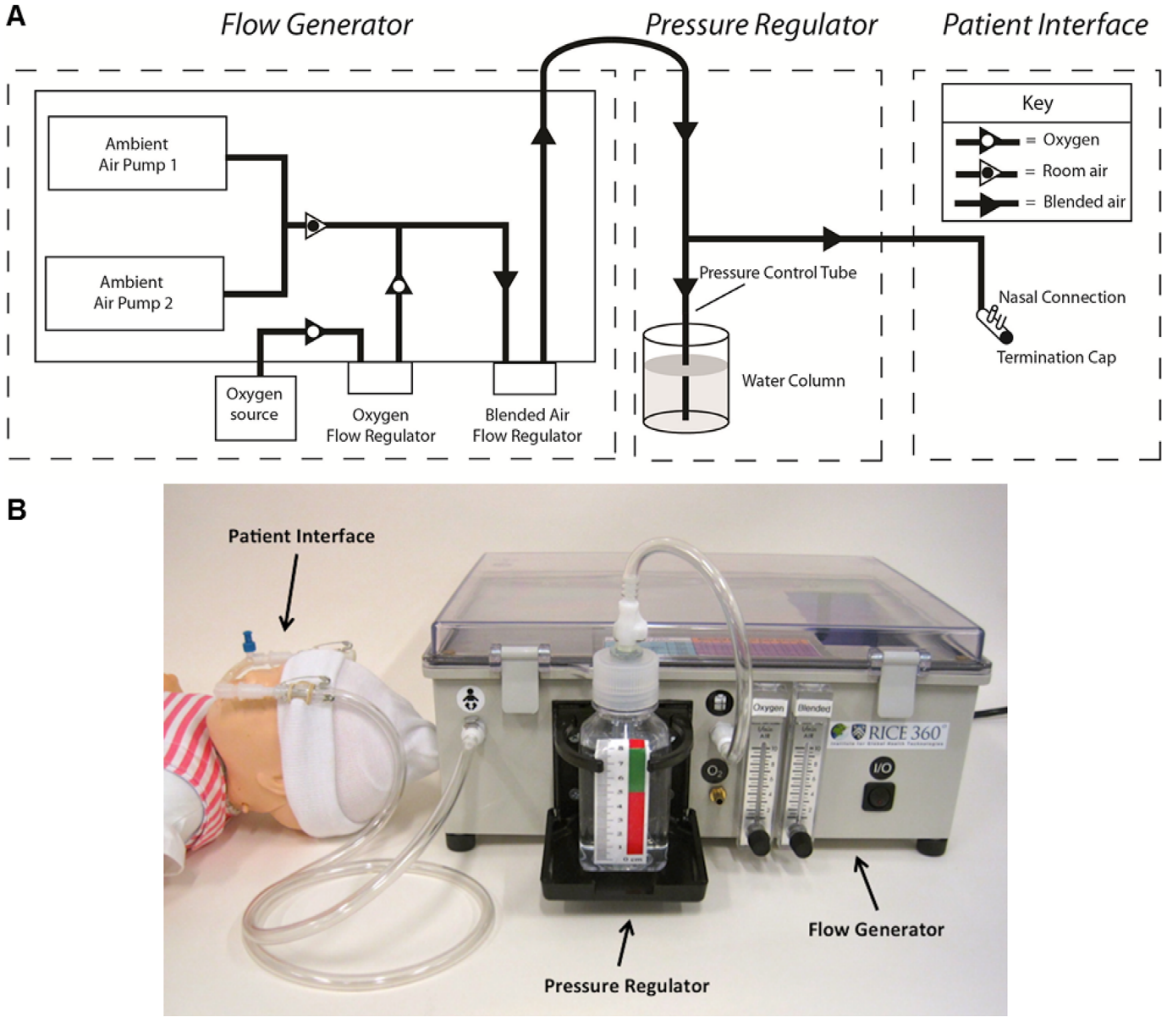
Block diagram and photograph of bCPAP system. The system consists of: (1) an adjustable flow generator; (2) a pressure-regulated delivery system; and (3) a patient interface. Flow is generated by two air pumps that can be blended with oxygen from a tank or concentrator. The total flow rate and fraction of oxygen delivered are controlled by two flow regulators. The output of the flow generator is connected to the pressure-regulated delivery system. Pressure is controlled by submerging a pressure control tube in a column of water; the mean pressure in the system is determined by the height of the water column. The patient interface is also connected to the pressure control tube, ensuring that the pressure in the patient interface and the pressure control tube are equivalent. The pressurized air mix is delivered to the patient's nostrils via a set of binasal prongs terminated at the distal end.

We designed a low-cost bubble Continuous Positive Airway Pressure (bCPAP) device for use in low-resource settings. The device delivers a source of continuous pressurized room air, which can be supplemented with oxygen from an oxygen concentrator or cylinder, if required. The blended, pressurized flow is delivered to the patient's nostrils via nasal prongs. Figure 1 shows a schematic diagram and photograph of the bCPAP device.

The device consists of three primary sub-systems: (1) an adjustable flow generator, (2) a pressure-regulated delivery system, and (3) a patient interface. The flow generator controls the flow rate and the mix of oxygen and air delivered to the patient; the delivery system controls the pressure delivered to the patient interface. The adjustable flow generator and pressure-regulated delivery system are placed next to the patient's bed, as shown in Fig. 1.

### Adjustable flow generator

Two diaphragm pumps are used to provide a continuous flow of room air through 0.25″ inner diameter vinyl tubing to a standard flow regulator, which can be adjusted to set the total flow rate provided to the delivery system. If required, an oxygen source can be connected to an input port in the flow generator. A second standard flow regulator is used to adjust the proportion of oxygen blended with room air. A look-up table on the device cover allows the user to quickly adjust the oxygen flow rate to the desired percentage of oxygen concentration for a given total flow rate.

### Pressure-regulated delivery system

The delivery system controls the pressure at which the blended air mix is delivered to the patient interface. The output of the flow generator enters the delivery system where it is connected in parallel to a pressure control tube and the patient interface. The distal tip of the pressure control tube is submerged in a column of water; the depth of water controls the pressure in the delivery system. The water bottle acts as a pressure relief valve; bubbles form as pressure in the system exceeds that set by the height of the water column.

### Patient Interface

The patient interface is designed to transfer pressure from the pressure control tube to the patient's airway. The pressurized air mixture is delivered to the patient's nostrils via a set of binasal prongs, terminated at the distal end with a short section of sealed tubing. Tubing leading to and from the prongs is attached to a stockinette hat with safety pins and elastic bands. This method of attachment holds the prongs securely in place, even when the baby moves, without adhesives, which could damage a baby's delicate skin [8].

### Pressure Testing Methodology

Pressure delivered by the bCPAP system was measured by blocking the outflow from the binasal prongs and connecting a pressure transducer (Model PX137-001DV, Omega Engineering, Inc.) just distal to the prongs. The device was set to deliver room air and prong pressure was measured for 60 seconds; data were collected at flow rates of 4, 6, 7, and 8 L/min and with the pressure control tube submerged in 4 and 6 cm of H_2_O. The bCPAP system was disassembled and reassembled, and the testing process was repeated 10 times. The average pressure was calculated; in addition, pressure minima and maxima were detected and the average peak pressures were calculated. As a reference standard, nasal prong pressures were obtained under the same conditions for a clinical bCPAP system used therapeutically at Texas Children's Hospital. Wall air was used as an adjustable flow generator, and an Airlife Infant heated wire circuit was used to deliver flow to the prongs and a pressure control system. Data from the two systems were compared for the same flow and pressure settings.

### Clinical Study

As part of a clinical study to evaluate the therapeutic performance of the bCPAP system at Queen Elizabeth Central Hospital, the device was used to treat a 6-month-old child with bronchiolitis and a neonate with respiratory distress due to congenital pneumonia. We monitored oxygen saturation, respiratory rate, work of breathing, and heart rate immediately before and after initiating therapy and then twice daily thereafter. Nasal saline drops were given every four hours during the duration of bCPAP therapy to prevent mucosal drying.

### Ethics Statement

The clinical study was reviewed and approved by the Institutional Review Boards at the College of Medicine Malawi, Rice University, and Baylor College of Medicine. Written informed consent was obtained for all participants. Written informed consent was obtained from the parents or guardians involved in this study.

## Results

**Figure 2:**
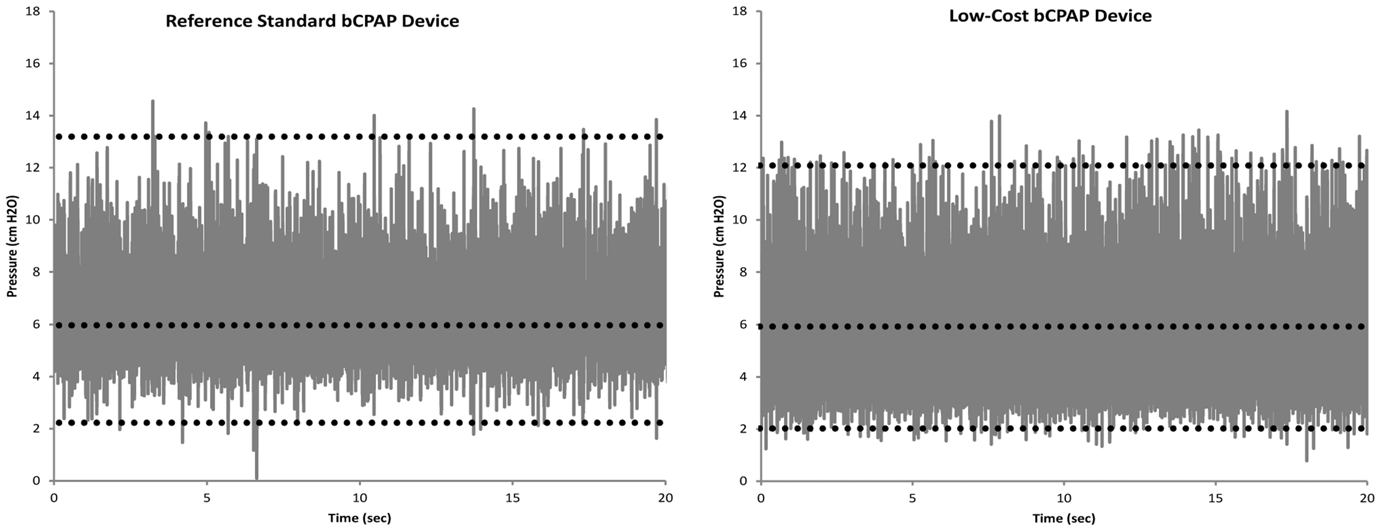
Pressure vs. time at the nasal prongs for two bCPAP devices. (Left) a reference standard bCPAP device used clinically in the US and (Right) the low-cost bCPAP device. Dotted lines show the mean and average peak pressures, averaged across 60 seconds of data collection. The pressure waveforms of the two devices are similar, indicating delivery of equivalent therapeutic pressure. In both devices, the mean pressure is controlled by adjusting the height of water in the pressure control tube, and the high frequency oscillations about the mean are associated with the formation of bubbles at the distal tip of the pressure control tube. There were no statistically significant differences between the pressures generated by the two devices (Student t-test, p<0.01).

Table 1 provides the operating specifications of the low-cost bubble CPAP device; the cost-of-goods to fabricate a single device at low production volume is approximately $350. Figure 2 compares the nasal prong pressure vs. time for the low-cost bCPAP device and the reference standard bCPAP device at a flow rate of 7 L/min with the pressure control tube submerged in 6 cm of H_2_O; the high frequency oscillations in pressure are associated with bubble formation at the distal tip of the pressure control tube. At these settings, both devices exhibit similar pressure waveforms, with an average pressure of 5.9 cm H_2_O, and average minimum and maximum peak pressures of 2.7 and 12.1 cm H_2_O for the low-cost bCPAP device, and an average pressure of 6.0 cm H_2_O, and average peak pressures of 3.3 and 13.2 cm H_2_O for the reference standard bCPAP device.

**Table 1:** bCPAP device operating specifications.

Parameter	Specification
Materials Cost	$350
Size	40 cm×32 cm×18 cm
Weight	1.5 kg
Pressure	0–8 cm H_2_O
Flow	0–10 L/min
Air/Oxygen Mix	40–60% Oxygen

**Figure 3:**
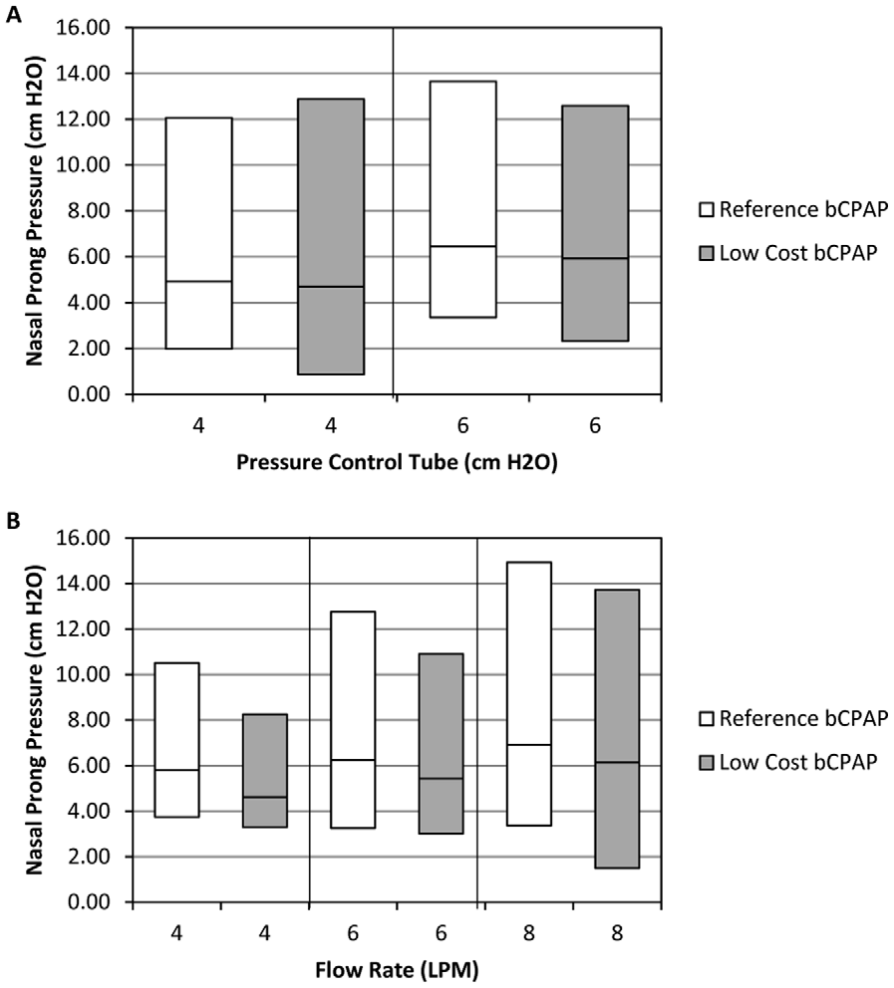
Comparison of reference standard and low-cost bCPAP output pressure under different flow and pressure settings. Each bCPAP system was assembled and nasal prong pressure was measured for 60 seconds of operation and mean pressures were calculated; results were then averaged for 10 independent trials of each system. (A) The mean pressure (mid-point of bar) and peak low and high pressures at a flow rate of 7 L/min at varying pressure settings. (B) The mean pressure (mid-point of bar) and peak low and high pressures at a pressure of 6 cm H_2_O and varying flow rates.

Figure 3 shows the average pressure and average peak pressures measured for both devices as the flow rate changes from 4 to 8 L/min and the distal tip of the pressure control tube is submerged in increasingly greater depths of H_2_O. Results reported are averaged across 10 independent measurements for the low-cost bCPAP and the reference standard device. Again, the devices show similar results, indicating that the devices deliver similar therapeutic pressures across a wide range of input parameters. Student t-tests were performed to test whether differences in the pressure data at each flow and pressure setting were statistically significant. There was no significant difference between the pressures generated by the two devices (p<0.01).

**Figure 4:**
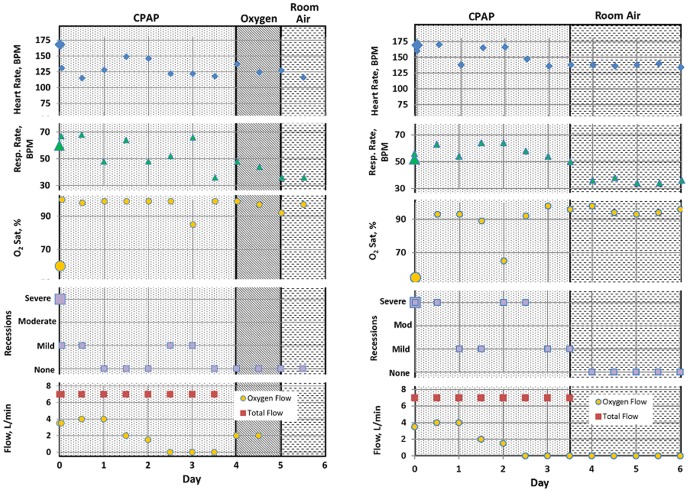
Vital signs for 6-month old patient with bronchiolitis (a) and a neonate with respiratory distress (b) immediately before and after initiation of bCPAP. (A) Time course immediately before treatment (large symbols) and after initiation of therapy (small symbols). The patient received CPAP treatment with gradually decreasing oxygen flow for 4 days, was then transitioned to nasal oxygen, and finally transitioned to room air. The patient was discharged on day 6. (B) Time course immediately before treatment (large symbols) and after initiation of therapy (small symbols). The patient received CPAP treatment for 3.5 days. The fraction of oxygen was gradually decreased to room air during the first 2 ½ days.

The low-cost bCPAP device was used to treat a 6-month-old baby admitted to Queen Elizabeth Central Hospital with bronchiolitis. Upon admission and immediately before undergoing bCPAP treatment, the child was unresponsive; her oxygen saturation was 60%, respiratory rate was 60 breaths per minute with severe recessions, and heart rate was 168 bpm. She was started on bCPAP therapy with a total flow rate of 7 L/min, 50% oxygen, and pressure of 6 cm H_2_O; Fig. 4a tracks changes in the child's vital signs before initiation of bCPAP therapy and over time after initiation of bCPAP therapy. One hour after starting bCPAP therapy, her oxygen saturation was 100%, respiratory rate was 67 with mild recessions, and heart rate was 131 bpm. Within 6 hours of initiating bCPAP, she was able to breast feed. She remained on bCPAP for 4 days; during this time the flow rate was 7 L/min, but the fraction of oxygen was gradually decreased to room air after 3 days. No evidence of mucosal drying or other complications were observed during bCPAP treatment. After discontinuation of bCPAP therapy, she was placed on nasal oxygen (2 L/min) for 1 day. Oxygen therapy was discontinued on day 5, and she was discharged on day 6.

The bCPAP device was also used to treat a full-term neonate with respiratory distress caused by congenital pneumonia. The baby's birth weight was 2.9 kg. His initial oxygen saturation was 55%, respiratory rate was 52 breaths per minute with severe recessions, and heart rate was 169 bpm. The baby was started on bCPAP therapy with a total flow rate of 7 L/min, 50% oxygen, and pressure of 6 cm H_2_O; Fig. 4b tracks changes in the baby's vital signs before initiation of bCPAP therapy and over time after initiation of bCPAP therapy. Within 4 hours of initiating bCPAP, the baby's oxygen saturation was 93%, respiratory rate was 63 breaths per minute, and the heart rate was 170 bpm. He remained on bCPAP for 3.5 days; during this time the flow rate was 7 L/min, but the fraction of oxygen was gradually decreased to room air after 2.5 days. No evidence of mucosal drying or other complications were observed during bCPAP treatment.

## Discussion and Conclusions

This low-cost bubble CPAP device delivers therapeutic pressure and flow equivalent to those of similar systems used in the developed world; however, the bCPAP device we have developed costs more than 15-times less. The device delivers 0–10 L/min of air flow and 0–8 cm water pressure and can be used to treat babies weighing up to 10 kg. It has been used to treat more than 100 infants and children in a low-resource setting. There have been no adverse events associated with the use of the bCPAP device. The low-cost device does not have all the features of bCPAP systems used in the developed world. Notably, the device does not heat or humidify the pressurized air mix delivered to the child. We have found that the use of nasal saline drops prevents mucosal drying without the risk of introducing pathogens into a child's airway when a ready supply of clean water for humidification may not be available. A rigorous clinical trial is required to fully evaluate this technology, but it holds promise as a method of successfully treating babies in need of respiratory support in low-resource settings.

To have global impact, it is not sufficient to simply develop an affordable, effective bCPAP system. In addition, staff must be trained to deliver bCPAP therapy and be able to access the ancillary equipment and disposable supplies necessary for effective bCPAP. Equipment and supplies include: a suction machine to clear nasal secretions, an oxygen concentrator or oxygen cylinder for supplemental oxygen, and nasal prongs, stockinettes, safety pins, and elastic bands. Some of these peripherals are available in Malawi. For example, the Government of Malawi, through its Child Lung Health Programme, provided oxygen concentrators, staff training, and supplies to all central and district hospitals to facilitate the sustainable delivery of oxygen to treat hypoxia [9]. At current market rates, the cost of disposable nasal prongs is likely to be a barrier to scale up of bubble CPAP in low-resource settings. In addition, parents must be willing for their newborns to receive bCPAP therapy when needed. This is one potential barrier to wide-scale dissemination in Malawi, for example, where many parents are reluctant to allow their children to receive any oxygen therapy because they associate the need for oxygen with a poor outcome [9].

A number of studies have shown that it is possible to successfully implement bCPAP in low-resource settings if devices are available [10]–[12]. Several low-cost bCPAP systems have been described recently [13]. Diblasi et al have described lower cost options for providing ventilatory support to neonates that could be implemented in low-resource settings if sources of air flow and oxygen are available [14]. A team from PATH and Hindu Rau Hospital is developing a low-cost bCPAP system for use in India, but it requires wall oxygen, which is not commonly available in district hospitals in Africa. East Meets West Foundation has developed a low-cost bCPAP system, which, in partnership with General Electric (GE), is being disseminated in Asia; at $2,800, this device is not affordable in district hospitals in Africa. There is still no bCPAP system available that meets the needs of most African rural hospitals.

While it is necessary to perform a more comprehensive assessment of the impact of bubble CPAP on reducing neonatal mortality due to RDS, recent studies suggest that the availability of bCPAP could have significant impact. A recent review article summarized historical declines in mortality due to RDS as new treatments were introduced in the United States (US), from 1903 to the present [4]. In the absence of treatment, neonatal RDS is almost always fatal [4]. The introduction of nasal oxygen, the current standard of care in central and district hospitals in Malawi, is thought to have improved survival rates to 25%; and it is speculated that the introduction of CPAP will increase survival rates to 70%. If this improvement in survival is realized, we estimate that on the African continent, where nearly one million babies die each year within a week of birth [15], providing bCPAP in central and district hospitals could prevent 178,000 early neonatal deaths.

## References

[1] World Bank (2006) Acute Respiratory Infections. In: Jamison DT, editor. Disease and Mortality in Sub-Saharan Africa. Washington (DC): World Bank Publications, 2006. pp. 149–162.

[2] Duke T (2005) Neonatal pneumonia in developing countries. Arch Dis Child Fetal Neonatal Ed 90: F211–F219. 15846010 10.1136/adc.2003.048108 PMC1721897

[3] Lawn JE, Kerber K, Enweronu-Laryea C, Cousens S (2010) 3.6 Million Neonatal Deaths – What is Progressing and What Is Not? Semin Perinatol 34: 371–386. 21094412 10.1053/j.semperi.2010.09.011

[4] Kamath BD, MacGuire ER, McClure EM, Goldenberg RL, Jobe AH (2011) Neonatal Mortality From Respiratory Distress Syndrome: Lessons for Low-Resource Countries. Pediatrics 127 6: 1139–1146. 21536613 10.1542/peds.2010-3212 PMC9923778

[5] March of Dimes, PMNCH, Save the Children, World Health Organization (2012) Born Too Soon: The Global Action Report on Preterm Birth. In: Howson CP, Kinney MV, Lawn JE, editors. Geneva: WHO Press. pp 2–3.

[6] Nowadzky T, Pantoja A, Britton JR (2009) Bubble continuous positive airway pressure, a potentially better practice, reduces the use of mechanical ventilation among very low birth weight infants with respiratory distress syndrome. Pediatr 123 6: 1534–1540. 10.1542/peds.2008-1279 19482765

[7] Watson AD (2010) Premarket Notification Decision for Fisher & Paykel Healthcare Bubble CPAP System. U.S. Food and Drug Administration: 510 (k) Number K100011. Available: http://tinyurl.com/k100011. Accessed 2012 Dec 6.

[8] Bonner KM, Mainous RO, Short M, Ikuta L (2008) The Nursing Care of the Infant Receiving Bubble CPAP Therapy. Adv Neonatal Care 8 2: 78–95. 18418205 10.1097/01.ANC.0000317256.76201.72

[9] Enarson P, La Vincente S, Gie R, Maganga E, Chokani C (2008) Implementation of an oxygen concentrator system in district hospital paediatric wards throughout Malawi. Bull World Health Organ 86 5 344–348. 18545736 10.2471/BLT.07.048017 PMC2647442

[10] Koyamaibole L, Kado J, Qovu JD, Colquhoun S, Duke T (2006) An Evaluation of Bubble-CPAP in a Neonatal Unit in a Developing Country: Effective Respiratory Support That Can Be Applied By Nurses. J Trop Pediatr 52 4: 249–253. 16326752 10.1093/tropej/fmi109

[11] Van Den Heuvel M, Blencowe H, Mittermayer K, Rylance S, Couperus A, et al (2011) Introduction of bubble CPAP in a teaching hospital in Malawi. Annals of Trop Pediatr 31 1: 59–65. 10.1179/1465328110Y.0000000001 21262111

[12] Urs PS, Khan F, Maiya PP (2009) Bubble CPAP - A Primary Respiratory Support for Respiratory Distress Syndrome in Newborn. Indian Pediatr 46: 409–411. 19179737

[13] Kaur C, Sema A, Beri RS, Pulivyel JM (2008) A Simple Circuit to Deliver Bubbling CPAP. Indian Pediatr 45: 312–314. 18451452

[14] Diblasi RM, Zignego JC, Smith CV, Hansen TN, Richardson CP (2010) Effective gas exchange in paralyzed juvenile rabbits using simple, inexpensive respiratory support devices. Pediatr Res 68 6: 526–30. 20814347 10.1203/PDR.0b013e3181f985f0

[15] World Health Organization (2006) Neonatal and Perinatal Mortality: Country, Regional and Global Estimates. Geneva: WHO Press. pp 22–57.

